# Anthropometric Measurements and Correlations to Glucometabolic and Cardiovascular Risk in Obese Patients Undergoing Gastric Bypass Surgery

**DOI:** 10.1155/2021/6647328

**Published:** 2021-07-16

**Authors:** Erica Aldenbäck, Hans-Erik Johansson

**Affiliations:** ^1^Bariatric Clinic, Department of Surgery, Falun Hospital, Falun, Sweden; ^2^Department of Public Health and Caring Sciences, Clinical Nutrition and Metabolism, Uppsala University, Uppsala, Sweden

## Abstract

Abdominal obesity is associated with hypertension, increased fasting glucose, HbA1c, and cholesterol. Body mass index (BMI) is frequently used to measure and define obesity and as inclusion criteria for bariatric surgery. Sagittal abdominal diameter (SAD) has been suggested to predict the amount of visceral fat, metabolic traits, and cardiometabolic risk superior to BMI. The aim was to test whether SAD has stronger correlations to glucometabolic traits compared to BMI. One hundred and fifty-five (108 women, 47 men) morbidly obese patients undergoing bariatric surgery were evaluated before (baseline), 6 and 12 months after Roux-en-Y gastric bypass (RYGBP). BMI was reduced from 43.7 kg/m2 (baseline) to 31.3 kg/m2 (12 months) and SAD from 32.6 to 23.2 cm (both *p*<0.001). SAD correlated with CRP (*p*=0.04), fasting glucose (*p*=0.008), HbA1c (*p*=0.016), triglycerides (*p*=0.017), systolic blood pressure (*p*=0.032), and vitamin D (*p*=0.027). BMI correlated with CRP (*p*=0.006), triglycerides (*p*=0.016), vitamin D (*p*=0.002), and magnesium (*p*=0.037). Despite RYGBP surgery, vitamin D was significantly increased. Liver enzymes were significantly lowered after RYGBP and the change over time in SAD correlated with gamma-glutamyltransferase. SAD was superior to BMI to predict glucose disturbance and dyslipidemia implying increased use of SAD as it is cost effective and simple to perform in the clinic and could be of value when considering patients for bariatric surgery.

## 1. Introduction

Obesity is an independent risk factor for type 2 diabetes mellitus (T2DM), metabolic syndrome, cardiovascular disease (CVD), and death [1]. Body mass index (BMI) is frequently used to measure and define obesity. BMI is also well established and often used in general as well as an inclusion criterion for bariatric surgery. Other measurements often used to characterize obesity are waist circumference, waist-to-hip ratio, sagittal abdominal diameter (SAD), and percentage of body fat calculated with magnetic resonance imaging or bioelectrical impedance. The ability to evaluate the visceral adipose tissue varies between methods [2]. Abdominal obesity is associated with metabolic syndrome, hypertension, higher fasting glucose, increased glycated hemoglobin (HbA1c), and higher cholesterol [3]. SAD has been suggested to predict the amount of visceral fat, metabolic traits, and cardio metabolic risk superior to BMI [4]. With the increasing number of overweight and obese patients, patients with the metabolic syndrome have also increased. The metabolic syndrome is a constellation of metabolic disturbances that are all risk factors for CVD [5]. The specific pathways leading to the metabolic syndrome is not yet fully known. There is a consensus that inflammatory pathways play a critical role in progression and development of the metabolic syndrome [6] with elevated acute-phase proteins such as C-reactive protein (CRP) and cytokines [7]. CRP has been shown to be an independent predictor of future cardiovascular events in metabolic syndrome patients [8]. Hypomagnesemia has also been suggested as a marker for the cascade that leads to the metabolic syndrome, as hypomagnesemia has been reported in patients with diabetes, obesity, and hypertension [9]. Magnesium is a cofactor involved in many enzymatic processes as well as in glucose metabolism, and T2DM is clearly associated with low magnesium and hypomagnesemia is associated with insulin resistance, inflammation, and increased risk for CVD [10, 11]. Haglin et al. have previously reported a significant increase in all-cause mortality when magnesium was added to traditional risk factors in patients with diabetes [12]. Nonalcoholic fatty liver disease (NAFLD) is also considered a manifestation of the metabolic syndrome and is associated with obesity and insulin resistance [13]. Higher concentrations of gamma-glutamyltransferase (GGT) and alanine aminotransferase (ALT) are associated with fibrosis and lobular inflammation of the liver [14]. NAFLD is also associated with higher GGT and ALT [15]. Framingham Heart Study showed that an augmentation in serum GGT predicts the onset of metabolic syndrome, incident CVD, and death, suggesting that GGT is a valuable marker of metabolic and cardiovascular risk [16]. Low 25-OH vitamin D (vitamin D) concentrations can be seen in obese individuals and are associated with CVD, metabolic syndrome, and T2DM [17]. Data regarding anthropometric measurements and glucometabolic status and cardiovascular risk are still few and divergent [18, 19]. The aim in this study was to test whether SAD is a better predictor of glucometabolic traits and cardiovascular risk compared to BMI and weight by analyzing glucose, HbA1_c_, inflammatory makers, lipids, magnesium, vitamin D, and liver enzymes in morbidly obese patients undergoing Roux-en-Y gastric bypass (RYGBP) surgery.

## 2. Method and Materials

### 2.1. Patients

This was a retrospective study of 1-year outcome of patients admitted during one year to a single center, Outpatient Clinic Obesity Care, Säter, Sweden. One hundred and fifty-five morbidly obese patients, 18 years or older, undergoing RYGBP surgery (108 women, 47 men), all Caucasians, and nonsmoking, were included. They were evaluated preoperatively (baseline), 6 months (1st follow-up) and 12 months (2nd follow-up) after RYGBP. Initially, a total of 198 consecutive preoperative evaluations were performed during one year regarding bariatric surgery. Finally, 168 patients were operated, and 155 underwent Roux-en-Y gastric bypass surgery with completed follow-ups. One patient underwent biliopancreatic diversion with duodenal switch and two patients underwent gastric sleeve operation. Thus, ten patients did not complete follow-ups. All of these patients were excluded from the study. Sixty one patients (39%) had T2DM and mean diabetes duration was 6.4 ± 5.6 years. Eighty-five (55%) patients were taking antihypertensive medication and 46 (30%) patients were on pharmacological treatment for dyslipidemia. The study was approved by the regional ethics review board at Uppsala University (register number 2017-108). Informed consent was obtained from all individual participants included.

### 2.2. RYGBP-Procedure

The RYGBP procedures were performed as previously described [20, 21]. All patients were given the same kind of dietary advice after surgery and were advised to take two tablets daily of oral supplement containing vitamins and minerals (MittVal Kvinna®) and an intramuscular injection of 1 mg cobalamin (vitamin B_12_) every third month. MittVal Kvinna® (per tablet) contains, e.g., vitamin D 7.5 *μ*g and magnesium 58 mg.

### 2.3. Test Procedures

All participants underwent physical examination and anthropometric measurements. Blood samples were collected, following an overnight fast and fluid consumption was restricted to a total of 500 ml of water 12 hours before examination. Routine blood tests were analyzed using Equalis and quality assured routine tests by analyzing plasma CRP, glucose, ApoA_1_, ApoB, triglycerides, ALT, GT, magnesium, and calcium in Abbott Architect ci 8200. B-HbA1c in Tosoh G8LA, S-vitamin D in Abbott Architect i2000SR, P-parathormone in Roche Cobas e411, and S-calcium (ionized) in Radiometer ABL 825 were all at the Department of Clinical Chemistry at Falun Hospital, Sweden.

### 2.4. Clinical Measurements

**Figure 1:**
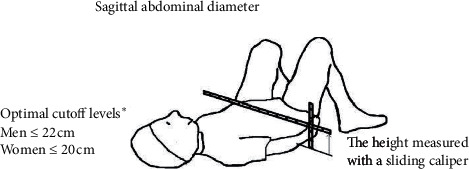
Sagittal abdominal diameter (SAD) measures abdominal fat. The distance between the examination couch and the height of the abdomen (cm) at level umbilical level is recorded in supine position. SAD cutoffs for an elevated cardiometabolic risk in men are ∼22 cm and in women ∼20 cm. ^*∗*^Sagittal abdominal diameter as a screening tool in clinical research: cutoffs for cardiometabolic risk. J Obes. 2010; 2010: 757939.

Blood pressure was measured on standardized sphygmomanometers. Weight (kg) and height (m) were measured on standardized calibrated scales and BMI (kg/m^2^) was calculated. SAD was recorded twice as the height of the abdomen (cm) at the umbilical level. This was done in supine position with a portable sliding beam caliper from the examination couch (Figure 1). Two measurements were taken by a physician. The mean value of the two measurements is presented. The same sliding beam caliper was used for the measurements and the patients were followed-up by the same physician. Optimal SAD cutoffs for an elevated cardiometabolic risk score in men are ∼22 cm and in women ∼20 cm [22].

### 2.5. Statistics

The same group was evaluated at three different time points: baseline, 6 months, and 12 months. Statistical analyses were made using paired *t*-test for parametric data and Wilcoxson for nonparametric data. Correlations were analyzed using Pearson's correlations coefficient for parametric data and Spearman's rank correlation coefficient was used for nonparametric data. *p* value of <0.05 was considered significant and data are presented as means (±2.5 SD). Analyses were made using JMP 5.0 (SAS, Texas).

## 3. Results

### 3.1. Baseline Data

Patient characteristics described at baseline and across the 12-month follow-up are shown in Table 1.

### 3.2. Follow-Up Data 12 Months after RYGBP

Over the 12-month period, there were significant mean reductions in BMI, weight, SAD, CRP, platelet counts, fasting glucose, HbA1_c_, ApoB/ApoA_1_, triglycerides, ALT, GGT, and systolic blood pressure and significant mean increases in vitamin D and magnesium (all *p* < 0.001) values presented in Table 1. In subanalyses of vitamin D in the group without vitamin D medication, by excluding 11 patients, the data were still robust. Despite RYGBP surgery, vitamin D increased significantly from 49.6 ± 17.4 nmol/l (baseline) to 63.3 ± 18.8 nmol/l (follow-up of 6 months) and further to 64.9 ± 21.5 nmol, (follow-up of 12 months), all *p* < 0.001 in patients without any pharmacological substitution except multivitamin (MittVal Kvinna®). In a subgroup of 61 individuals with T2DM, magnesium increased from 0.78 ± 0.07 to 0.82 ± 0.07 mmol/l (*p* < 0.001), whereas in nondiabetic patients, it was unchanged, 0.82 mmol/l ±0.06 (*p*=0.137), over the period. There was no difference in renal function regarding plasma creatinine in between the groups.

### 3.3. Correlations

At baseline SAD significantly correlated with CRP (*r* = 0.54, *p*=0.04), fasting glucose (*r* = 0.22, *p*=0.008), HbA1c (*r* = 0.24, *p*=0.016), triglycerides (*r* = 0.21, *p*=0.017), systolic blood pressure (*r* = 0.18, *p*=0.032), and vitamin D (*r* = −0.18, *p*=0.027) but not with remaining analyzed markers (Table 2).

At 12-month follow-up SAD significantly correlated with fasting glucose (*r* = 0.28, *p*=0.002), HbA1c (*r* = 0.24, *p*=0.011), ApoB/A1 (*r* = 0.33, *p* < 0.001), triglycerides (*r* = 0.37, *p* < 0.001), and vitamin D (*r* = -0.15, *p*=0.023).

At baseline BMI correlated with CRP (*r* = 0.24, *p*=0.006), triglycerides (*r* = 0.20, *p*=0.016), vitamin D (*r* = -0.27, *p*=0.002), and magnesium (*r* = −0.17, *p*=0.037) (Table 2).

At 12-month follow-up BMI correlated with fasting glucose (*r* = 0.24, *p*=0.003), ApoB/A1 (*r* = 0.32, *p* < 0.001), triglycerides (*r* = 0.30, *p* ≤ 0.001), and vitamin D (*r* = −0.20, *p*=0.016).

At baseline, weight correlated with triglycerides (*r* = 0.23, *p*=0.004) and vitamin D (*r* = −0.23, *p*=0.007) and at 12 months only with triglycerides (*r* = 0.30, *p* < 0.001).

Analyzing the in change over the 12-month period in percent (Δ%) SAD correlated with the change in GGT (*r* = 0.25, *p*=0.010) and triglycerides (*r* = 0.23, *p*=0.0021). BMI correlated with the change in triglycerides (*r* = 0.22, *p*=0.015).

### 3.4. Pharmacological Treatment

Sixty-one (39%) patients had type T2DM. At baseline 38 (*n*) patients were on medication with metformin and 23 (*n*) patients were on other antidiabetic drugs such as insulin, sulfonylureas, DPP-4 inhibitors, and GLP-1 analogues or lifestyle intervention. Diabetes remission was observed in 45 of 61 patients (74%). After 12 months, 10 patients were still on medication (5 on insulin and 5 on metformin) and the rest performed only lifestyle interventions. SAD correlated with T2DM remission (*p*=0.047) at 12 months but BMI did not (*p*=0.104). The numbers of patients with pharmacological treatment for hypertension at baseline versus 12-month follow-up were 85 (55%) and 62 (40%), respectively, and patients with treatment for dyslipidemia were 46 (30%) and 19 (12%), respectively.

## 4. Discussion

In this study we performed anthropometric measurements and blood samples during one year, to determine time-dependent changes in morbidly obese patients undergoing RYGBP in glucometabolic markers and their correlations with anthropometric measurements of obesity and particularly abdominal obesity. SAD correlated at baseline, in these obese patients, with CRP, triglycerides, and systolic blood pressure and to glucometabolic parameters such as glucose and HbA1_c_. SAD emerged as a better predictor of glucose metabolism than BMI and weight which was in accordance with data from NHANES investigation of US Adults [23].

Over the time RYGBP surgery induced large improvements in metabolic parameters; e.g., glucose, HbA1_c,_ lipids, liver enzymes, blood pressure, and correlations were found to be components of the metabolic syndrome. The metabolic syndrome is a constellation of metabolic disturbances that are all risk factors for CVD [24]. SAD correlated at baseline with three of its components (glucose or HbA1_c_, blood pressure, and triglycerides) whereas BMI correlated with one (triglycerides). SAD could be considered as a valuable marker for metabolic risk [19, 25] and a key predictor of abdominal obesity regarding cardiovascular morbidity and mortality [4]. NAFLD is also tightly connected to the metabolic syndrome and GGT is associated with liver inflammation and evaluated predictor of metabolic and cardiovascular risk [16]. Over the period, a clear decrease of GGT concentrations was observed and a significant correlation over time, per percent, was shown to the change in SAD.

Magnesium is in several ways involved in glucose metabolism and insulin resistance; it is a cofactor for GLUT-4 mediated glucose uptake in the peripheral tissue and a regulator of enzymes involved in gluconeogenesis in the liver [26] and reduces the effects of glucagon which upregulate liver glucose production [27]. In this study, the subgroup of 61 patients with T2DM, magnesium increased significantly along with improved glucometabolic status and 45 out of 61 patients (74%) showed remission of T2DM. At 12 months, there was a correlation observed between SAD and T2DM remission but not with BMI. In the nondiabetic group, the magnesium concentrations were unchanged over the period. The impact on magnesium could be of significance since magnesium is a major risk factor for all-cause mortality in T2DM patients [12] and even low-to-normal magnesium concentrations are associated with higher mortality in patients with T2DM [26, 28]. Magnesium plays also a protective role in the development of T2DM [28]. Vitamin-D is more unusual and less validated marker regarding glucometabolic status with association to insulin sensitivity. Vitamin D deficiency and obesity are associated with cardiovascular disease, metabolic syndrome, and T2DM [17]. Shangara et al. have reported a strong interaction between vitamin D with BMI with impact on fasting glucose and HOMA [29]. Both BMI and SAD showed correlations with vitamin D at baseline and follow-up. The improvement in glucometabolic status in these obese patients along with weight reduction was accompanied by increased vitamin D concentrations over the period. In obesity, lower vitamin D concentrations are common, and in this study an increase was observed without changes in calcium and parathormone with no vitamin D substitution except multivitamin which was somewhat unexpected as the early literature often emphasized the risk of deficiency [30]. Possible, a release of this fat-soluble vitamin D from the fat tissue could be involved. Low concentrations should be avoided as vitamin D is involved in bone metabolism and may accelerate the development of T2DM in obese individuals [17]. SAD has been evaluated against waist circumference and BMI to the oral glucose tolerance test (direct measure of glucose metabolism) along with fasting glucose, HbA1_c_ and HOMA-IR in overweight patients and SAD turned out to be a better predictor of glucose metabolism, risk for prediabetes and T2DM compared to waist circumference and BMI [19]. This did not apply to normal-weight adults. We found similar findings in glucose parameters regarding SAD in comparison to BMI. Within this morbidly, obese population SAD was superior to BMI in correlations to cardiovascular risk factors and components of the metabolic syndrome. This implies that the inclusion criteria for bariatric surgery could possibly be widened. As of now, patients with BMI over 40 kg/m^2^ are generally included without any obesity-related comorbidities, but patients with BMI 35–39.9 kg/m^2^ need to have a comorbidity to be included for bariatric surgery. In light of our study, this could be questioned, since higher BMI within this obese group does not necessarily imply higher cardiovascular and metabolic risk. SAD was superior in this obese group and should be considered as an addition to the BMI inclusion criterion. Regarding anthropometric measurements, we did not have waist circumference. The size of this study is small, simple statistical analysis is used, and the correlations are at times not strong; thus, larger studies are needed to confirm our results or trends. Still data and conclusions are in accordance with larger studies such as NHANES investigation of US adults [23] with the distinction that we evaluated morbidly obese patients.

## 5. Conclusion

Anthropometric measurements in morbidly obese individuals showed associations to markers of abdominal obesity and glucometabolic traits. In this study, SAD was superior to BMI and weight in evaluating glucose disturbance and dyslipidemia (components of metabolic syndrome), implying increased use of SAD as it is cost effective and simple to perform in the clinic, and SAD could also be of value to evaluate and include patients for bariatric surgery.

## Figures and Tables

**Table 1:** Baseline characteristics, 6-month and 12-month follow-up data of 155 morbidly obese patients who underwent Roux-en-Y gastric bypass surgery.

	Baseline	6 months	12 months	*p* for trend
Gender (women/men)	108/47	—	—	—
Age (years)	45.5 (11.0)	—	—	—
Height (cm)	169.4 (8.8)	168.8 (8.4)	168.7 (8.5)	NS
Weight (kg)	126.0 (21.4)	93.9 (17.0)	89.3 (18.4)	<0.001
BMI (kg/m^2^)	43.7 (6.3)	32.7 (5.0)	31.3 (5.8)	<0.001
Sagittal diameter (cm)	32.6 (3.9)	24.1 (3.4)	23.2 (4.2)	<0.001
B-Hb (g/l)	143 (11.1)	140 (11.3)	138 (10.5)	<0.001
CRP (mg/l)	7.6 (4.3)	5.2 (1.6)	5.4 (2.1)	<0.001
Platelets counts (x10^9^/l)	302 (70)	268 (69)	255 (61)	<0.001
P-glucose (mmol/l)	7.5 (2.9)	5.7 (1.0)	5.8 (1.5)	<0.001
HbA1_c_ (mmol/mol)	47.5 (15.6)	37.6 (8.2)	36.9 (8.9)	<0.001
P-ApoB/ApoA_1_	0.71(0.20)	0.55 (0.17)	0.52 (0.15)	<0.001
P-triglycerides (mmol/l)	2.01 (1.25)	1.27 (0.59)	1.16 (0.56)	<0.001
S-25-OH-vit D (nmol/l)	48.8 (17.1)	64.1 (19.5)	65.3 (21)	<0.001
P-parathormone (pmol/l)	7.8 (2.1)	7.5 (4.1)	8.0 (5.6)	NS
S-calcium (mmmol/l)	2.30 (0.09)	2.34 (0.08)	2.33 (0.09)	NS
P-magnesium (mmol/l)	0.80 (0.06)	0.82 (0.06)	0.82 (0.06)	<0.001
P-ALT (*μ*kat/l)	0.74 (0.34)	0.63 (0.26)	0.65 (0.40)	<0.001
P-GGT (*μ*kat/l)	0.71 (0.46)	0.43 (0.49)	0.44 (0.51)	<0.001
SBP (mmHg)	135.8 (18.7)	126.2 (16.6)	129.0 (14.9)	<0.001
DBP (mmHg)	81.6 (10.3)	77.1 (9.9)	77.1 (10.1)	<0.001

Data shown are arithmetic means (±SD). *BMI:* body mass index, *CRP:* C-reactive protein, *GGT:* gamma-glutamyltransferase, *ALT:* alanine aminotransferase, *SBP:* systolic blood pressure, and *DPP:* diastolic blood pressure. *P:* plasma and *S:* serum.

**Table 2:** Correlations (*r*): BMI and SAD at baseline and 12 months. R is not shown for nonsignificant correlations.

	BMI correlations baseline	SAD correlations baseline	BMI correlations 12 months	SAD correlations 12 months
CRP (mg/l)	*r* = 0.24^∗∗^	*r* = 0.54^*∗*^	ns	ns
Platelets counts (×10^9^/l)	ns	ns	ns	ns
P-glucose (mmol/l)	ns	*r* = 0.22^∗∗^	*r* = 0.24^∗∗^	*r* = 0.28^∗∗^
HbA1_c_ (mmol/mol)	ns	*r* = 0.24^∗∗^	ns	*r* = 0.24^∗∗^
P-ApoB/ApoA_1_	ns	ns	*r* = 0.32^∗∗∗^	*r* = 0.33^∗∗∗^
P-triglycerides (mmol/l)	*r* = 0.20^*∗*^	*r* = 0.21^*∗*^	*r* = 0.30^∗∗∗^	*r* = 0.37^∗∗∗^
S-25-OH-vit D (nmol/l)	*r* = −0.27^∗∗^	*r* = −0.18^*∗*^	*r* = −0.20^*∗*^	*r* = −0.15^*∗*^
P-magnesium (mmol/l)	*r* = −0.17^*∗*^	ns	ns	ns
P-ALT (*μ*kat/l)	ns	ns	ns	ns
P-GGT (*μ*kat/l)	ns	ns	ns	ns
SBP (mmHg)	ns	*r* = 0.18^*∗*^	ns	ns
DBP (mmHg)	ns	ns	ns	ns

*BMI:* body mass index, *SAD:* sagittal abdominal diameter, *CRP:* C-reactive protein, *GGT:* gamma-glutamyltransferase, *ALT:* alanine aminotransferase, *SBP:* systolic blood pressure, *DBP:* diastolic blood pressure, *P:* plasma, and *S:* serum. Significance is indicated by “^*∗*^” (*p* < 0.05), “^∗∗^” (*p* < 0.01), and “^∗∗∗^” (*p* < 0.001). *ns:* nonsignificant (*p*-value).

## Data Availability

The data used to support the findings of this study are available from the corresponding author upon request.
